# Prevalence of intraoperative and postoperative iatrogenic mandibular fractures after lower third molar extraction: A systematic review

**DOI:** 10.4317/jced.58390

**Published:** 2022-01-01

**Authors:** Maria-Antonieta Guillaumet-Claure, Ana-María Juiz-Camps, Cosme Gay-Escoda

**Affiliations:** 1 MD, DDS. Master’s Degree Programme in Oral Surgery (EFHRE International University/FUCSO); 2 DDS. Fellow of the Master of Oral Surgery and Implantology. School of Dentistry of the University of Barcelona, Spain; 3 MD, DDS, PhD, MS, EBOS, OMFS, Chairman and Professor of Oral and Maxillofacial Surgery, School of Dentistry, Barcelona. Director of the Master’s Degree Programme in Oral Surgery and Implantology (EHFRE International University/ FUCSO). Coordinator/Researcher of the IDIBELL Institute. Head of the Oral Surgery, Implantology and Maxillofacial Surgery Department of the Teknon Medical Center, Barcelona, Spain

## Abstract

**Background:**

The surgical extraction of the lower third molars is one of the most common procedures in oral surgery, and this surgical operation can cause intra- and postoperative complications such as pain, trismus, bleeding, infection, oedema, inferior alveolar nerve injuries, displacement of teeth to neighbouring spaces and mandibular fractures. The aim of this systematic review is to report the prevalence of mandibular fractures that occur intra- and postoperatively in patients who have undergone surgical removal of the lower third molar.

**Material and Methods:**

An electronic database search for articles published in Cochrane, PubMed (MEDLINE) and Scopus was conducted using the key words “Molar, Third”; “Mandibular Fractures”; “Molar Third, Removal”; “Molar Third, Complications”; “Dental Extractions, Complications”; “Mandibular Fractures, Third molar removal”. The inclusion criteria were articles including at least 10 patients and were published in English in the last 10 years. The exclusion criteria were nonhuman studies and case reports.

**Results:**

Postoperative mandibular fractures after 3MI occur more frequently in male patients between the ages of 40 and 60 and are caused by premature chewing force. The parameters that most frequently characterise mandibular fractures at the mandibular angle are deeply impacted lower third molars, Class II and III, B and C, according to the Pell & Gregory classification system, mesioangular according to the Winter’s classification, and are located on the left mandibular side..

**Conclusions:**

Mandibular fractures can be predicted with adequate preoperative planning for each case and identify the related risk factors for this complication.

** Key words:**Molar, Third; Mandibular Fractures; Molar Third, Removal; Molar Third, Complications; Dental Extractions, Complications; Mandibular Fractures, Third molar removal.

## Introduction

Surgical extraction of the lower third molars is one of the most common procedures in oral surgery (1-4). This surgical procedure may be accompanied by intra- and postoperative complications such as pain, trismus, bleeding, infection, oedema, inferior alveolar nerve injuries, displacement of teeth to neighbouring spaces and mandibular fractures (1-6). The mandible has some weak areas that are less resistant to fractures such as the mandibular angle, the condyle, the mandibular symphysis, the body and coronoid process (7,8). The specific bone anatomy of the gonial angle with its location between the ascending branch and the mandibular body as well as its association with the inclusion of the lower third molar makes it one of the most frequent fracture sites (40%) (8.9).

Mandibular fractures are some of the most severe lower third molar complications that can occur. This complication has a relatively low rate of incidence, ranging from 0.0034% to 0.075% for lower third molar extractions (1), and with similar percentages in the studies published by Joshi *et al*. (2), Boffano *et al*. (3) and Bodner *et al*. (5). In the study published by Grau-Manclús *et al*. (6), they report a narrower range from 0.0033% to 0.0046%, and Ethunandan *et al*. (4), establish an incidence of 0.00033% and 0.0049%. Postoperative fractures are the most common, with an incidence ranging from 0.0042% (7) to 0.0046% (10) in contrast with intraoperative fractures, which vary between 0.0033% (7) and 0.0036% of cases (6).

Mandibular fractures can occur intraoperatively or as a late complication during the postoperative course, generally within the first 4 weeks after surgery (2,5). Inadequate management of surgical instruments, the application of excessive force, incorrect surgical technique, underestimating the difficulty of the extraction, not performing the correct odontosection of the lower third molar and performing extensive ostectomies may be some of the causes of iatrogenic fractures (3,7,11). In addition, areas of bone weakened by pathological processes such as cystic and/or malignant injuries, osteoporosis, osteomyelitis or medication-related osteonecrosis of the jaw caused by bisphosphonates are other possible causes of pathological fractures (3,10,11), with these making up less than 2% of all total mandibular fractures (3).

According to previously published studies, the cause of this complication is multifactorial, and it has been demonstrated there are different factors that may contribute to and increase the risk of this event. These include the age and gender of the patient, the position and angulation of the lower third molar, a possible previous infectious pathology associated with the tooth to be extracted, as well as postoperative care like consuming a soft food diet and moderate chewing (1,2,5).

The aim of this systematic review is to report the prevalence of intra- and postoperative mandibular fractures in patients who underwent surgical removal of lower third molars. The secondary objectives are to establish what the risk factors for this complication are and the most common location of these fractures.

In order to accomplish these objectives, a PICO question was formulated: What is the frequency, location and risk factors associated with the appearance of intra- or postoperative mandibular fractures in patients who require the removal of third molars?

 

## Material and Methods

**Figure 1:**
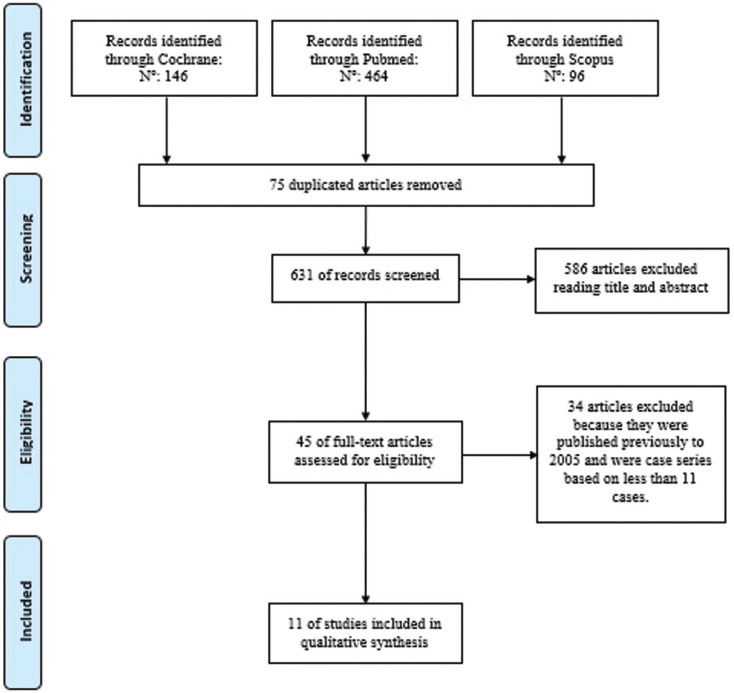
PRISMA flow diagram (12).

An electronic database search for articles published in Cochrane, PubMed (MEDLINE) and Scopus with the key words “Molar, Third”; “Mandibular Fractures”; “Molar Third, Removal”; “Molar Third, Complications”; “Dental Extractions, Complications”; “Mandibular Fractures, Third molar removal”, following the PRISMA flow diagram (Fig. 1) (12) was carried out.

The inclusion criteria were systematic reviews and meta-analyses of cases of intra- or postoperative mandibular fractures after lower third molar removal, which included at least 10 cases, were published between 2005 and 2021, written in English, and analysed risk factors related to this surgical complication (Table 1). Articles published before 2005 and based on nonhuman studies in which mandibular fractures unrelated to the extraction of the lower third molar were reported (5,14,16) and series of less than 10 cases were excluded.

**Table 1:**
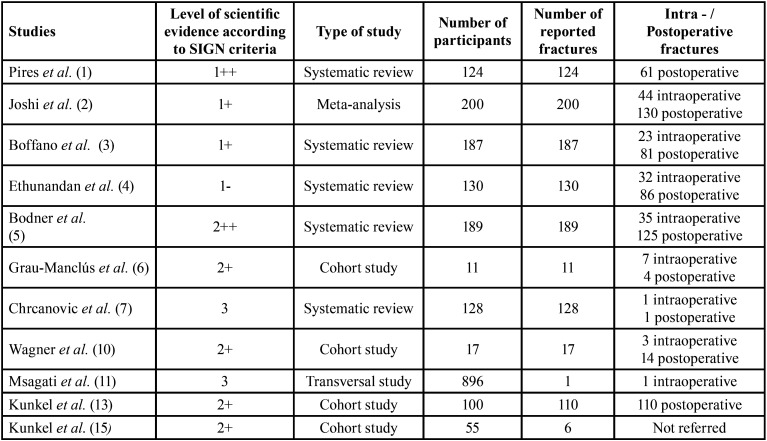
Studies selection according to the level of scientific evidence stablished by SIGN criteria (17).

The selected articles, classified according to their level of scientific evidence according to the SIGN criteria (17), are reflected on Table 1. They were analysed by two independent reviewers by first analysing the titles and summaries and then through full text examination (Table 1). Data collection was carried out independently, including copies of the original articles and data extraction in order to analyse demographic variables such as age and gender, aetiology, the intra- or postoperative period of the fracture (Table 2,  Table 2 cont.), anatomical conditions like the position of the lower third molar (according to Pell & Gregory’s classification) (18), the angulation (Winter’s classification) (19), the degree of impaction (total or partial impaction), as well as the location of the fracture in the mandibular anatomy (Table 3,  Table 3 cont.).

**Table 2:**
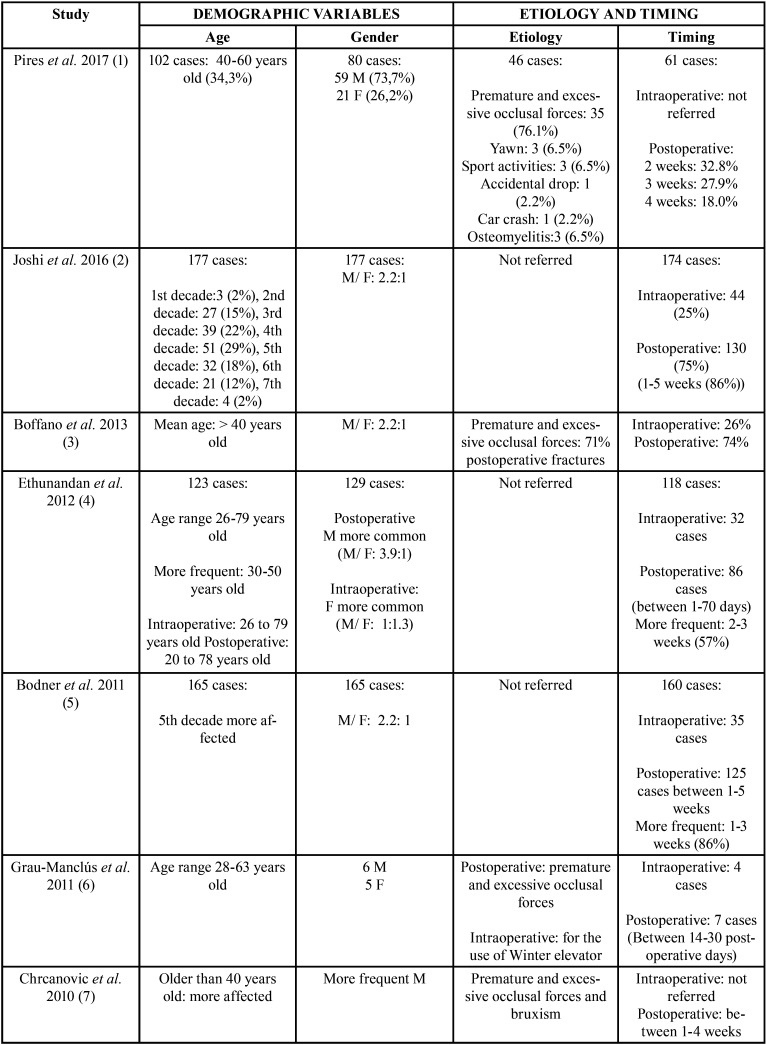
Demographic variables (age and gender) (M: male gender F: female gender), etiology and timing of mandibular fractures (intraoperative or postoperative) (weeks (wk)) (1-7,10,11,13,15).

**Table 2 cont.**
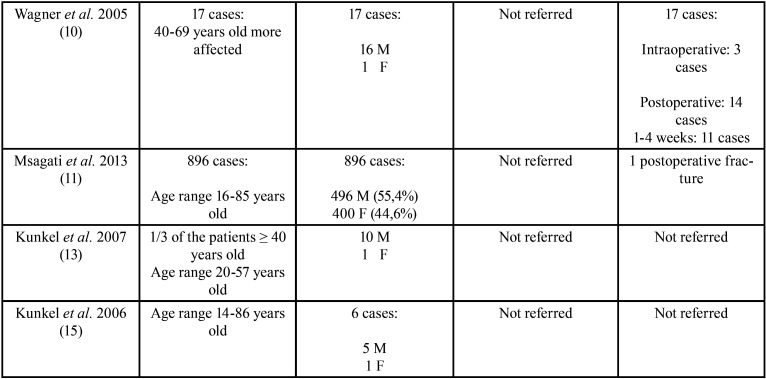
Demographic variables (age and gender) (M: male gender F: female gender), etiology and timing of mandibular fractures (intraoperative or postoperative) (weeks (wk)) (1-7,10,11,13,15).

**Table 3:**
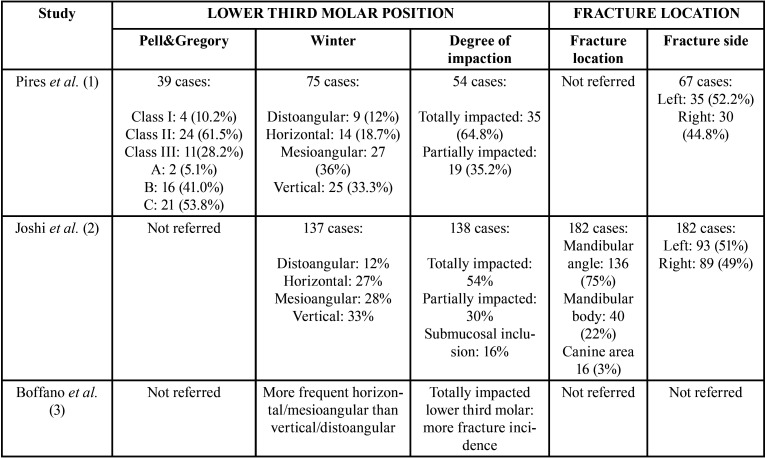
Lower third molar position and description of the location of the fractures (anatomic location and affected side) (1-7,10,11,13,15).

**Table 3 cont.**
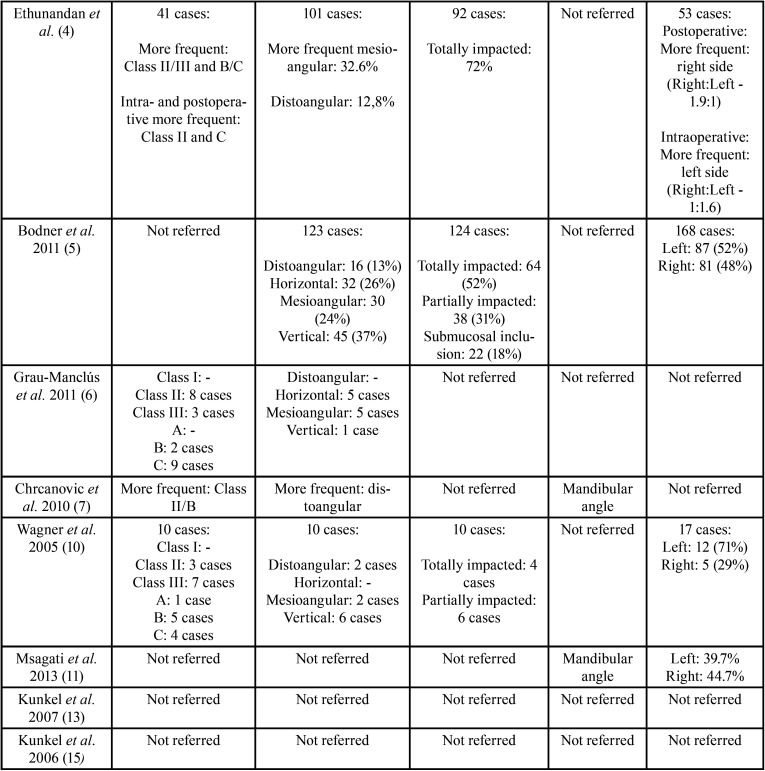
Lower third molar position and description of the location of the fractures (anatomic location and affected side) (1-7,10,11,13,15).

## Results

706 studies were obtained from the initial search after eliminating duplicate articles, analysing the title and those which did not meet the previously established inclusion criteria. Of these, the full texts of 45 articles were analysed for their eligibility. Finally, an additional 34 articles were eliminated for not meeting the previously established inclusion criteria leaving 11 studies (1-7,10,11,13,15) (Table 2, 2 cont.) that are included in this systematic review whose objective is to analyse mandibular fractures as intra-or postoperative complications as a result of third molar extraction (Fig. 1).

Demographic variables, age and gender were documented in all the studies that were included (1-7,10,11,13,15). They report that the highest frequency of mandibular fractures occurs in male patients (91.6%). Only one study established that intraoperative fractures occur more frequently in female patients, in contrast with postoperative fractures, which occur more commonly in men (4). The most affected patients were over 40 ranging between 40 and 60 years of age (66.66%) (1-5,7,10,13) and in 4 studies (6,11,15), the age range varied greatly from 16 to 86 years of age (33.33%) (Table 2,  Table 2 cont.).

With regard to aetiology and the timing of the fractures, it was observed that the intraoperatively inappropriate use of surgical instruments (6), premature postsurgical occlusal force (80%) (1,3,6,7) and bruxism (7), are the main aetiological factors associated with these fractures. It should be noted that these factors were not reported in 8 of the 11 studies included in the analyses (2,4,5,10,11,13-15). The time of fracture onset was analysed in 9 of the included studies (75%) (1-5,6,7,10,11); the most frequent were postoperative fractures (88.88%). Among the postoperative fractures, it was observed that the most prevalent postoperative time range was between 1 and 4 weeks after lower third molar removal (66%) (2,4,5-7,10) (Table 2,  Table 2 cont.).

In relation to the position of the lower third molar, its location according to Pell & Gregory’s classification (18), the angulation according to Winter’s classification (19), and the degree of impaction (total or partial) were analysed (Table 3,  Table 3 cont.). According to Pell & Gregory’s classification, fractures occur more frequently in lower third molars class II (85.33%) and III, and at position B (66.66%) or C (1,4,6,7,10). The least frequent position was Class I and position A (1). With regard to angulation, the mesioangular position (45.45%) was associated with a higher number of fractures, followed by the vertical (36.36%), horizontal (18.18%) and distoangular position (9.09%) (1-5,6,7,10). With respect to degree of impaction, completely impacted lower third molars are more likely to be associated with the appearance of fracture events (71.42%) (1-5,10).

The location of this type of complication was more frequently observed on the left side of the jaw (85.7%) (1,2,4,5,10). The study published by Ethunandan *et al*. (4), mentions that the highest frequency of fractures on the left side was intraoperative in contrast with the right side, which is associated more often with fractures in the postoperative course. Furthermore, a higher frequency of fractures occurred at the mandibular angle level, followed by the mandibular body and the canine area (2,7,11) (Table 3,  Table 3 cont.).

## Discussion

Mandibular fractures related with the extraction of lower third molars are one of the most severe complications that can occur intra- or postoperatively. It can occur as an immediate or a late complication, generally within the first 4 weeks after the removal of 3MI (2-7,10). According to *Pi*res *et al*. (1), the greatest period of risk is during the second and third postoperative weeks, since the granulation tissue in the alveolus is being replaced by connective tissue and the resistance of the mandibular bone is decreased during this time

With regard to age and gender, 91.6% of fractures occurred in male patients (1-3,5,11) over 40 years old, with a range established between 40 and 60 years of age (1,7,10,13). According to Bodner *et al*. (5) and Özçakir-Tomruk *et al*. (31), this may be due to a delay in the maturation phase during the bone regeneration period and the weakening of bone tissue associated with a reduction in bone elasticity during the aging process starting in the fourth decade of life. Likewise, Perry and Golberg (30) highlight a delay in the bone granulation phase in older patients where two thirds of the socket are not filled with osteoid material, thereby causing a decrease in the resistance of the mandibular bone.

Pires *et al*. (1) mention that the decrease in bone elasticity and the appearance of osteoporosis in elderly patients may be another reason. Elderly patients experience greater thinning of the periodontal ligament and the incidence of ankylosis also increases which can increase the difficulty in removing the lower third molars, creating a considerable need for ostectomies which facilitate the chances of a possible fracture. With regard to gender, it has been noted that males have greater muscular strength which favours the appearance of excessive traction force during the postoperative course (32-34).

Fractures that occur during surgery are less frequent than postoperative ones (5,7,10). Intraoperative factors related with this complication are the inappropriate use of surgical instruments, incorrect surgical techniques in which excessive force is exerted (6), a mesioangular position of the lower third molar (4,8,30,35,36) probably due to the fact that mesioangular and vertical angulations are more prevalent in the general population as highlighted by Morales-Trejo *et al*’s study (37), and a relationship with the anterior zone of the mandibular ascending ramus type II and III, and Pell & Gregory’s depths B and C (18) (1-7,10,14). Pires *et al*. (1) claim that this is probably attributed to a greater degree of difficulty in extracting the lower third molar, making more extensive ostectomies necessary. These authors also mention the relationship between postoperative mandibular fractures and a history of pericoronitis, which could be related to the fact that recurrent or chronic infections can contribute to decalcification and, therefore, a greater probability of fracture (1). Ethunandan *et al*. (4) and Grau-Manclús *et al*. (6) established the use of Winter’s drift as an inappropriate instrument, since it has a shorter stem and a thicker handle making it easy to apply excessive force with the first application of the first-class lever.

Perry and Goldberg (30) mention that a bone area with a weakened structure is created after extracting the lower third molars making the appearance of this type of complication more likely. Though intraoperative fractures are less frequent, 7 of 11 mandibular fractures were inoperable in the Grau-Manclús’ *et al*. study (6). This may be because five of the intraoperative fractures occurred in lower third molars associated with dentigerous cysts and odontogenic tumours. These alterations can cause significant weakening of the bone, particularly in the region of the mandibular angle. It can therefore be concluded that there is a relationship between the presence of pathological bone changes and the subsequent appearance of fractures.

In the study published by Wagner *et al*. (10), of 17 fractures, 12 occurred on the left side which may be attributed to worsened view of the surgical field from the surgeon’s right side, resulting in a less extensive ostectomy. With regard to the degree of impactation, Chrcanovic and Custodio (7), relate it to a surgical approach that includes quite extensive ostectomies, thereby favouring fractures at the level of the mandibular angle (11,36-38).

According to Perry and Goldberg (30), the risk of fracture is higher in the first 2to 3 weeks of the postoperative course therefore fractures occurring in the immediate or late postoperative period are more frequent than intraoperative ones (1-7,10,11). Premature and excessive occlusal force have been associated with this complication. Pires *et al*. (1), affirm that the masticatory force necessary to break down food before swallowing it can generate a considerable amount of tension in the weakened mandibular region after lower third molar extraction. Perry and Goldberg *et al*. (30) reported that mandibular fractures occurred in patients who did not correctly follow the postoperative instructions for a soft food diet. Libersa *et al*. (8), claim that patients abandoned the soft diet and resumed daily activities and physical sport in the first 2-3 weeks of the postoperative course as their general condition and symptoms improved making these sorts of fractures are more frequent. Joshi *et al*. (2) discusses the possibility that postoperative fractures may be incomplete intraoperative fractures, which may have exceeded the tolerance of stress limits in the weeks after the extraction, given that patients felt better and painful symptoms had almost disappeared at the end of the second week.

The risk of impaired sensitivity of the inferior alveolar nerve should also be analysed when fractures during the extraction of the lower third molar occur. Boffano *et al*. (40) analyse the incidence of inferior alveolar nerve injuries after the incidence of mandibular fractures at the body level, the angle or the mandibular ascending branch or after traumatic events such as car accidents or other traumatic events in 325 hospital patients. Patients with condylar fractures and comminuted or multiple fractures were excluded. Finally, 79 patients (24.3%) with inferior alveolar nerve alterations were included in the study. These sensory alterations were analysed by means of a two-point discrimination test, using the non-fractured side of the same patient as a control. Patients diagnosed with hypoesthesia, paraesthesia, or anaesthesia were included. No statistically significant relationship was found between the occurrence of changes in sensitivity as a result of an inferior alveolar nerve injury in relation to a mandibular fracture, but its association with traumatic injuries caused by trauma in the mandibular region was significant.

Tay *et al*. (41), described neurosensorial alterations that occurred in 80 patients who had experienced mandibular fractures as a result of traumatic events; 49.3% of the fractures caused an alteration in the sensitivity of the inferior alveolar nerve, while fractures at the condylar level (13%) were not associated with episodes of sensory alterations. The overall prevalence of inferior alveolar nerve injuries was 33.7%, before the surgical approach to mandibular fractures, and 53.8% after fracture reduction and fixation using titanium miniplates. According to this study, one of the risk factors associated with the development of these injuries after a mandibular fracture was related to the type of surgical approach used to reduce the fracture and the separation distance between the reduced fragments. According to this study, a distance greater than 1 mm results in a 27% increase in the probability of postoperative sensory alterations. The factors determining the recovery time after an alteration in the sensitivity of the inferior alveolar nerve include the type of surgical approach used to reduce and fix the fracture for its consolidation, the location of the fracture in the region of the mandibular angle, the time elapsed between the fracture event and its surgical reduction, as well as the patient’s age. Therefore, mandibular fractures in the posterior mandibular region exhibit a high prevalence of associated neurosensorial disorders (56.2%).

Risk of injury to the inferior alveolar nerve should be considered for all patients who have had a mandibular fracture, either in the intra- or postoperative course after lower third molar removal, resulting from the displacement of both fractured fragments and during the surgical reduction approach. These patients should be advised that these sensory changes can occur in the postoperative period, and that in order to accelerate and promote sensory recovery, it is necessary to implement a pharmacological treatment associated with the application of low-level laser therapies (42,43).

Finally, the results obtained in this systematic review of 11 studies should be relativized by taking into account that 3 of the included studies (5,6,11) had a low level of evidence according to the Scottish Intercollegiate Guidelines Network criteria for evaluating scientific evidence (SIGN) (17), which are reported on Table 1. The exclusion criteria also had case series of less than 10 patients due to their limited level of evidence. In addition, it has been observed in the studies we analysed, that there are numerous parameters that were not reported for analysis, such as the aetiology of the intra- or postoperative fracture, characteristics of the extracted lower third molar (in terms of their three-dimensional position, such as the degree of impactation) in addition to the location of the fracture itself. 

## Conclusions

Considering the limitations of this study, it can be concluded that a comprehensive preoperative analysis of the frequency of different risk factors related to mandibular fractures occurring after the removal of the 3MI is necessary. It should include demographic variables like age and gender, the planning of the surgical technique according to the position, angulation and degree of impaction of the 3MI. There is an increased risk of intraoperative and postoperative mandibular fractures after the extraction of 3MI in male patients over 35 years old, with fully impacted 3MI that are mesio-angulated and classified as II or III and B or C, according to Pell & Gregory and Winter classification systems. The mandibular angle is the most frequent location of intraoperative and postoperative fractures, followed by the mandibular body and the canine area.

Moreover, intraoperative mandibular fractures occur more frequently on the left mandibular side, unlike postoperative ones, which occur more frequently on the right side. A detailed clinical and radiographic study of each case and employing adequate surgical techniques are the most relevant strategies in preventing the appearance of mandibular fractures after the extraction of 3MI.

## References

[1] Pires WR, Bonardi JP, Faverani LP, Momesso GAC, Muñoz XMJ, Silva AFM (2017). Late mandibular fracture occurring in the postoperative period after third molar removal: Systematic review and analysis of 124 cases. Int J Oral Maxillofac Surg.

[2] Joshi A, Goel M, Thorat A (2016). Identifying the risk factors causing iatrogenic mandibular fractures associated with exodontia: A systemic meta-analysis of 200 cases from 1953 to 2015. Oral Maxillofac Surg.

[3] Boffano P, Roccia F, Gallesio C, Berrone S (2013). Pathological mandibular fractures: A review of the literature of the last two decades. Dent Traumatol.

[4] Ethunandan M, Shanahan D, Patel M (2012). Iatrogenic mandibular fractures following removal of impacted third molars: An analysis of 130 cases. Br Dent J.

[5] Bodner L, Brennan PA, McLeod NM (2011). Characteristics of iatrogenic mandibular fractures associated with tooth removal: Review and analysis of 189 cases. Br J Oral Maxillofac Surg.

[6] Grau-Manclús V, Gargallo-Albiol J, Almendros-Marqués N, Gay-Escoda C (2011). Mandibular fractures related to the surgical extraction of impacted lower third molar: A report of 11 cases. J Oral Maxillofac Surg.

[7] Chrcanovic BR, Custódio AL (2010). Considerations of mandibular angle fractures during and after surgery for removal of third molars: A review of the literature. Oral Maxillofac Surg.

[8] Libersa P, Roze D, Cachart T, Libersa JC (2002). Inmediate and late mandibular fractures after third molar removal. J Oral Maxillofac Surg.

[9] Lim HY, Jung TY, Park SJ (2017). Evaluation of postoperative complications according to treatment of third molars in mandibular angle fracture. J Korean Assoc Oral Maxillofac Surg.

[10] Wagner KW, Otten JE, Schoen R, Schmelzeisen R (2005). Pathological mandibular fractures following third molar removal. Int J Oral Maxillofac Surg.

[11] Msagati F, Simon EN, Owibingire S (2013). Pattern of occurrence and treatment of impacted teeth at the Muhimbili National Hospital, Dar es Salaam, Tanzania. BMC Oral Health.

[12] Moher D, Liberati A, Tetzlaff J, Altman DG, PRISMA Group (2009). Preferred reporting items for systematic reviews and meta-analyses: The PRISMA statement. PLoS Med.

[13] Kunkel M, Kleis W, Morbach T, Wagner W (2007). Severe third molar complications including death - lessons from 100 cases requiring hospitalization. J Oral Maxillofac Surg.

[14] Al-Belasy FA, Tozoglu S, Ertas U (2009). Mastication and late mandibular fracture after surgery of impacted third molars associated with no gross pathology. J Oral Maxillofac Surg.

[15] Kunkel M, Morbach T, Kleis W, Wagner W (2006). Third molar complications requiring hospitalization. Oral Surg Oral Med Oral Pathol Oral Radiol Endod.

[16] Boffano P, Ferretti F, Giunta G (2012). Surgical removal of a third molar at risk for mandibular pathologic fracture: Case report and clinical considerations. Oral Surg Oral Med Oral Pathol Oral Radiol.

[17] (2011). SIGN 50: A guideline developer's handbook. 2nd ed. Scottish Intercollegiate Guidelines Network (SIGN).

[18] Pell GJ, Gregory GT (1933). Impacted mandibular third molars: Classifications and modified technique for removal. Dent Digest.

[19] Winter GB (1926). Impacted mandibular third molars.

[20] Szucs A, Buitár P, Sándor GK, Barabás J (2010). Finite element analysis of the human mandible to assess the effect of removing an impacted third molar. J Can Dent Assoc.

[21] Woldenberg Y, Gatot I, Bodner L (2007). Iatrogenic mandibular fracture associated with third molar removal. Can it be prevented?. Med Oral Patol Oral Cir Bucal.

[22] Cankaya AB, Erdem MA, Cakerer S, Cifter M, Oral CK (2011). Iatrogenic mandibular fracture associated with third molar removal. Int J Med Sci.

[23] Özcakir-Tomruk C, Arsaln A (2012). Mandibular angle fractures during third molar removal: A report of two cases. Aust Dent J.

[24] Dos Santos Silva W, Silveira RJ, de Araujo Andrade MGB, Franco A, Silva RF (2017). Is the late mandibular fracture from third molar extraction a risk towards malpractice? Case report with the analysis of ethical and legal aspects. J Oral Maxillofac Res.

[25] Correa AP, Perez L, Ramalho-Ferreira G, Ferreira S, Ávila F, de Oliveira I (2014). Unerupted lower third molar extractions and their risks for mandibular fracture. J Craniofac Surg.

[26] Xu JJ, Teng L, Jin SL, Lu JJ, Zhang C (2014). Iatrogenic mandibular fracture associated with third molar removal after mandibular angle osteotectomy. J Craniofac Surg.

[27] Kao YH, Huang IYE, Chen CM, Wu CW, Hsu KJ, Chen CM (2010). Late mandibular fracture after lower third molar extraction in a patient with Stafne bone cavity: A case report. J Craniofac Surg.

[28] Steinberg JP, Hirsch EM, Olsson AB, Olsson AB (2013). Functionally stable fixation for an infected mandibular angle fracture associated with third molar extraction during pregnancy. J Craniofac Surg.

[29] Komerik N, Draduman Al (2008). Mandibular fracture 2 weeks after third molar extraction. Dent Traumatol.

[30] Perry PA, Goldberg MH (2000). Late mandibular fracture after third molar surgery: A survey of Connecticut oral and maxillofacial surgeons. J Oral Maxillofac Surg.

[31] Özçakir-Tomruk C (2012). Mandibular angle fractures during third molar removal: A report of two cases. Aust Dent J.

[32] Miyaura K, Matsuka Y, Morita M, Yamashita A, Watanabe T (1999). Comparison of biting forces in different age and sex groups: A study of biting efficiency with mobile and non-mobile teeth. J Oral Rehabil.

[33] Lim HY, Jung TY, Park SJ (2017). Evaluation of postoperative complications according to treatment of third molars in mandibular angle fracture. J Korean Assoc Oral Maxillofac Surg.

[34] Patel N, Kim B, Zaid W (2016). A Detailed analysis of mandibular angle fractures: Epidemiology, patterns, treatments, and outcomes. J Oral Maxillofac Surg.

[35] Gbotolorun OM, Olojede AC, Arotiba GT, Ladeinde AL, Akinwande JA, Bamgbose BO (2007). Impacted mandibular third molars: Presentation and postoperative complications at the Lagos University Teaching Hospital. Nig Q J Hosp Med.

[36] Krimmel M, Reinert S (2000). Mandibular fracture after third molar removal. J Oral Maxillofac Surg.

[37] Morales-Trejo B, Rocha-Navarro ML, Acosta-Veloz AL, Juárez-Hernández A (2012). Class, type and position of 9148 surgically removed third molars in 3206 patients: A retrospective study. Med Oral Patol Oral Cir Bucal.

[38] McNamara Z, Findlay G, O'Rourke P, Batstone M (2016). Removal versus retention of asymptomatic third molars in mandibular angle fractures: A randomized controlled trial. Int J Oral Maxillofac Surg.

[39] Osborn TR, Frederickson G, Small IA, Torgerson TS (1985). A prospective study of complications related to mandibular third molar surgery. J Oral Maxillofac Surg.

[40] Boffano P, Roccia F, Gallesio C, Karagozogly K, Forouzanfar T (2014). Inferior alveolar nerve injuries associated with mandibulat fractures at risk: A two-center retrospective study. Craniomaxillofac Trauma Reconstr.

[41] Tay AB, Lai JB, Lye KW, Wong WY, Nadkarni NV, Li W (2015). Inferior alveolar nerve injury in trauma-induced mandible fractures. J Oral Maxillofac Surg.

[42] Miloro M, Criddle TR (2018). Does low-level laser therapy affect recovery of lingual and inferior alveolar nerve injuries?. J Oral Maxillofac Surg.

[43] Ozen T, Orhan K, Gorur I, Ozturk A (2006). Efficacy of low level laser therapy on neurosensory recovery after injury to the inferior alveolar nerve. Head Face Med.

